# Ovariectomy-induced hormone deprivation aggravates Aβ_1-42_ deposition in the basolateral amygdala and cholinergic fiber loss in the cortex but not cognitive behavioral symptoms in a triple transgenic mouse model of Alzheimer’s disease

**DOI:** 10.3389/fendo.2022.985424

**Published:** 2022-10-11

**Authors:** Szidónia Farkas, Adrienn Szabó, Bibiána Török, Csenge Sólyomvári, Csilla Lea Fazekas, Krisztina Bánrévi, Pedro Correia, Tiago Chaves, Dóra Zelena

**Affiliations:** ^1^ Institute of Physiology, Medical School, University of Pécs, Centre for Neuroscience, Szentágothai Research Centre, Pécs, Hungary; ^2^ Laboratory of Behavioral and Stress Studies, Institute of Experimental Medicine, Budapest, Hungary; ^3^ János Szentágothai School of Neurosciences, Semmelweis University, Budapest, Hungary

**Keywords:** Alzheimer’s disease, hormone deprivation, ovariectomy, cognitive function, anxiety, estrogen, cholinergic neurons

## Abstract

Alzheimer’s disease is the most common type of dementia, being highly prevalent in elderly women. The advanced progression may be due to decreased hormone synthesis during post-menopause as estradiol and progesterone both have neuroprotective potentials. We aimed to confirm that female hormone depletion aggravates the progression of dementia in a triple transgenic mouse model of Alzheimer’s disease (3xTg-AD). As pathological hallmarks are known to appear in 6-month-old animals, we expected to see disease-like changes in the 4-month-old 3xTg-AD mice only after hormone depletion. Three-month-old female 3xTg-AD mice were compared with their age-matched controls. As a menopause model, ovaries were removed (OVX or Sham surgery). After 1-month recovery, the body composition of the animals was measured by an MRI scan. The cognitive and anxiety parameters were evaluated by different behavioral tests, modeling different aspects (Y-maze, Morris water maze, open-field, social discrimination, elevated plus maze, light–dark box, fox odor, operant conditioning, and conditioned fear test). At the end of the experiment, uterus was collected, amyloid-β accumulation, and the cholinergic system in the brain was examined by immunohistochemistry. The uterus weight decreased, and the body weight increased significantly in the OVX animals. The MRI data showed that the body weight change can be due to fat accumulation. Moreover, OVX increased anxiety in control, but decreased in 3xTg-AD animals, the later genotype being more anxious by default based on the anxiety z-score. In general, 3xTg-AD mice moved less. In relation to cognition, neither the 3xTg-AD genotype nor OVX surgery impaired learning and memory in general. Despite no progression of dementia-like behavior after OVX, at the histological level, OVX aggravated the amyloid-β plaque deposition in the basolateral amygdala and induced early cholinergic neuronal fiber loss in the somatosensory cortex of the transgenic animals. We confirmed that OVX induced menopausal symptoms. Removal of the sexual steroids aggravated the appearance of AD-related alterations in the brain without significantly affecting the behavior. Thus, the OVX in young, 3-month-old 3xTg-AD mice might be a suitable model for testing the effect of new treatment options on structural changes; however, to reveal any beneficial effect on behavior, a later time point might be needed.

## 1 Introduction

Alzheimer’s disorder (AD) is the most common type of dementia, which is among the top 10 leading causes of death in the world (1, 2). It is characterized by disturbances of memory, attention, and sleep (1, 3). The patients often have difficulties in their daily life due to their impaired behavioral abilities (4). Morphologically, amyloid plaques [formed by amyloid-β 1-42 (Aβ_1-42_)] and hyperphosphorylated tau aggregates appear in the hippocampus, cortex, and amygdala, brain areas that are critical in cognitive and emotional function (5, 6).

Plenty of risk factors have been identified regarding AD. These can be lifestyle related, like diet, physical activity, and environmental conditions, or medical factors, like obesity and cardiovascular conditions (1). However, the three major risk factors are age, gender, and genetical mutations (7–9). It is well known that the incidence of AD is increasing with age, but it is also important to note that women represent 70% of the patients (10). The increasing female prevalence among elderly can be due to hormonal change during menopause (11, 12). Namely, the low levels of sex steroids, like 17β-estradiol (E2) and progesterone (PG), may have an important role in the pathomechanism (13). Indeed, both E2 and PG play a pivotal role in neuroprotection, thereby improving cognitive function, memory, attention, synaptic plasticity, spine density, and dendrite formation (14–17). The loss of the ovarian hormones can affect these functions, and also increase the appearance of amyloidogenic markers, aggravating the progression of AD (18–20). Beside the natural decrease in ovarian steroids during menopause, the surgical removal of the gland in younger generation may also have detrimental effect on their cognitive capabilities (21, 22). It is estimated that, in USA, 100,000 cases of dementia may be attributable annually to bilateral oophorectomy (23). This later state can be modeled by ovariectomy (OVX) in animals (24, 25).

AD can also be characterized by genetical mutations, leading to family accumulations. Research has identified five main “AD genes”: apolipoprotein E (ApoE) ϵ4 allele, amyloid precursor protein (APP), presenilin-1 (PSEN1), presenilin-2 (PSEN2), and microtubule-associated protein tau (MAPT). These genes may contribute to the formation of amyloid plaques, leading to memory loss and behavioral changes (8, 26–33), as well as to different tauopathies such as AD (34, 35). Genetic animal models were generated based on these human mutations. The triple transgenic mouse (3xTg-AD), bearing the humanoid mutation of APP, PSEN1, and tau, is widely used and well characterized (36–38). This mouse strain develops AD-like structural (amyloid plaques and hyperphosphorylated tau) and behavioral (progressive cognitive decline) symptoms.

The most relevant and affected neurocircuit in AD patients is the cholinergic system (39, 40), most of all the basal forebrain cholinergic (BFC) neurons (41, 42), being the main therapeutic target (43). The cholinergic neurons from the medial septum (MS), nucleus basalis magnocellularis (NBM), and substantia innominata complex are highly affected in AD, and also express E2 receptors (44–48), proving the importance of sexual steroids in the pathophysiology of the disease. OVX may decrease, while E2 treatment normalizes the number of cholinergic neurons in the BFC, as well as the length and branching of these neurons (49–51). In the 3xTg-AD mouse model, a cholinergic decline was also discovered, showing the loss of ChAT immunoreactive neurons in the MS and in the vertical limb of the diagonal band of Broca (52, 53).

Based on the important role of sexual steroids in neuronal health and their role in mental diseases, we aimed to investigate the aggravating effect of hormone deprivation induced by bilateral OVX on AD-related somatic, behavioral, and histological changes in the 3xTg-AD mice. The lack of E2 and PG may anticipate difficulties in cognitive function and anxiety-related behavior, perturbs somatic characteristics (like body weight or body fat ratio), and assumes morphological changes on amyloid deposition and in the cholinergic system. To test this hypothesis, the following concepts were used: (I) As OVX is often accompanied by body weight increase (54), and uterus weight decrease (55, 56), we were concentrating on these somatic parameters mainly to confirm the effectiveness of the OVX surgery. (II) The major symptom of dementia is cognitive disability; therefore, we used behavioral tests measuring (57) (i) short-term memory [Y-maze, often used in AD testing (58); based on spontaneous exploration of the mice]; (ii) social discrimination (SD); (iii) spatial memory [Morris water maze (MWM) as the gold standard in AD research (59, 60); also known as avoidance-based complex association]; (iv) reward-based simple association [operant conditioning (OC)]; and (v) punishment-based simple association [conditioned fear test (CFT)]. (III) As anxiety is often comorbid with AD (61, 62), and is a core symptom during menopause, or after OVX (63), we tested these symptoms by (i) elevated plus maze (EPM), as a gold standard in anxiety research (64), showing changes during the menstrual cycle (65); (ii) light–dark box (LD) test, which utilizes the fear from open, light spaces, similarly to EPM; and (iii) fox odor test (FOT), measuring the innate fear from a predator odor. (IV) At the structural level, we were concentrating on Aβ accumulation as well as cholinergic cell and fiber loss.

## 2 Materials and methods

### 2.1 Mouse strains

Three-month-old 3xTg-AD [B6;129-Tg(APPSwe,tauP301L)1Lfa Psen1tm1Mpm/Mmjax] mice and their control strains (C57BL6/J) were used (66). This age corresponds to young adult humans without hormonal disturbances. The 3xTg-AD animals were homozygotes for three AD-related human-based genetic mutations: PSEN1, APPSwe, and tauP30IL (36–38). We maintained the colony by breeding homozygous mice to each other. Only females were used in this experiment. All animals were bred and housed at the Institute of Experimental Medicine, Budapest, Hungary. The mice were maintained under reversed light–dark cycle (lights off at 8:00 a.m., lights on at 8:00 p.m.) and provided with standard mice chow [without estrogen-free dietary restrictions (67)] and water *ad libitum*. The animal rooms have a temperature of 22 ± 2°C and a relative humidity of 55 ± 10%. All tests were approved by the local committee of animal health and care (PE/EA/918-7/2019) and performed according to the European Communities Council Directive recommendations for the care and use of laboratory animals (2010/63/EU).

### 2.2 Experimental design

**Figure 1:**
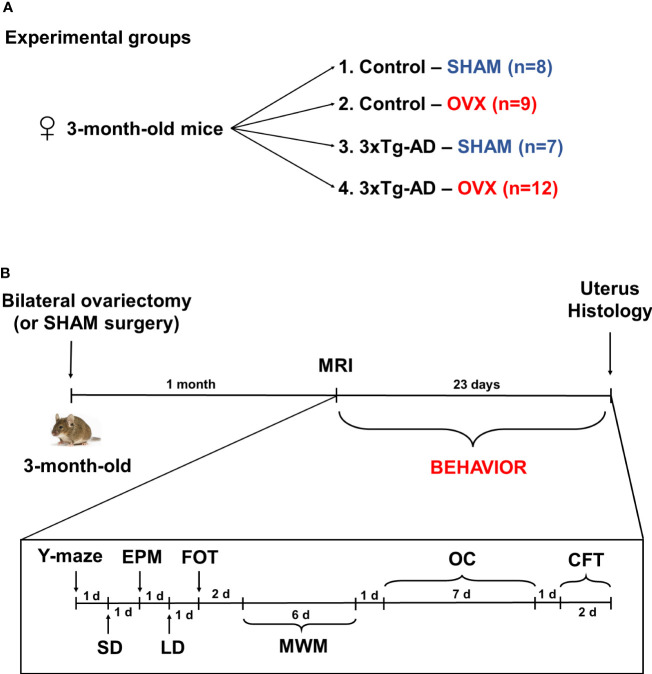
Experimental design. **(A)** Three-month-old female 3xTg-AD and C57BL6/J mice were used and divided into the following groups: (1) Control-Sham (*n* = 8), (2) Control-OVX (*n* = 9), (3) 3xTg-AD-Sham (*n* = 7), and (4) 3xTg-AD-OVX (*n* = 12). **(B)** Chronological order of experimental procedures. On 3-month-old mice, a bilateral ovariectomy (OVX) or Sham surgery was performed, then after 1 month, magnetic resonance imaging (MRI) measurements were conducted followed by behavioral experiments in this order: Y-maze, social discrimination (SD), elevated plus maze (EPM), light–dark box (LD), fox odor test (FOT), Morris water maze (MWM), operant conditioning (OC), and conditioned fear test (CFT). The duration and time between different tests are marked in the chronological axis as days (d). At the end of the experiment, uterus and brain were dissected for histological staining. OVX, ovariectomy; 3xTg-AD, triple transgenic mouse model of Alzheimer’s disorder.

Mice were ovariectomized (OVX) or Sham operated without removing the ovaries (Sham), under ketamine–xylazine anesthesia (dose: 125 mg/kg ketamine and 25 mg/kg xylazine dissolved in 0.9% saline, administered in 10 ml/kg concentration intraperitoneally). During surgery, the animals were divided into the following four groups: (1) Control-Sham (*n* = 8), (2) Control-OVX (*n* = 9), (3) 3xTg-AD-Sham (*n* = 7), and (4) 3xTg-AD-OVX (*n* = 12) (**Figure 1A**; the unequal animal numbers are due to surgical-related loss). Two series were conducted; each contained all four groups. After 1 month, a magnetic resonance imaging (MRI) measurement was performed. During this period, the ovarian hormones were supposed to disappear [maximal luteinizing hormone levels can be detected at this point (68)] and enough time has passed for the development of supposed behavioral changes. Then, the following behavioral test battery was used: Y-maze, SD, EPM, LD, FOT, MWM, OC, and CFT, with at least 24-h rest between the different tests (**Figure 1B**). The order of the tests was chosen from the milder stressors (5–10 min single test) to more burdensome ones (through restricted diet in OC till foot shock in CFT). All behavioral tests were performed during the first half of the active (dark) cycle (between 9:00 a.m. and 2:00 p.m.). At the end of the experiments, animals were sacrificed, and brains were dissected and post-fixed in 4% PFA for 24 h, dehydrated in 30% sucrose solution for 24 h, and then 30-µm-thick slices were made with a freezing microtome (Leica SM2010 R). Uterus dissection and weighting were also performed to validate the success of the OVX. Due to technical reasons (e.g., missing video recording and loss of brain slide during staining) in some experiments, data from one to two animals are missing.

### 2.3 Magnetic resonance imaging measurements

**Figure 2:**
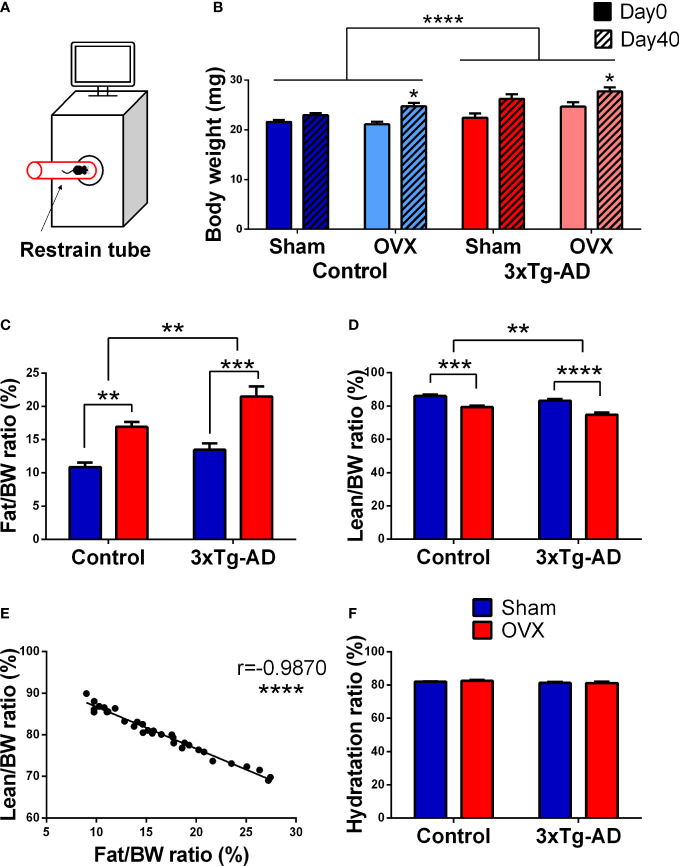
Magnetic resonance imaging (MRI) measurements. **(A)** Representative figure of the procedure. Animals were placed in a restrained tube, then inserted into an EchoMRI™-700 machine for approximately 1 min. **(B)** Body weight change of the animals from day 0 to day 40. 3xTg-AD animals were heavier than their controls (*p *< 0.0001). OVX induced body weight increase, irrespective of the genotype (*p* = 0.007). **(C)** Body fat percentage at 1-month post-surgery. 3xTg-AD animals had higher body fat percentage [Fat/Body weight (BW)*100] (*p* = 0.0034), which was aggravated by OVX in both genotypes (*p *< 0.0001). **(D)** Body lean percentage 1-month after surgery. A decrease in the body lean ratio [Lean/Body weight (BW)*100] was detected after OVX (*p *< 0.0001), and between the two genotypes (*p* = 0.0016). **(E)** Correlation between body fat and lean ratio. A negative and significant correlation was seen between the body fat and lean ratio (*p *< 0.0001). **(F)** Hydration ratio of the different animal groups. The hydration ratio (HR = ((total water − free water)/lean)*100) was normal in all animals (80% ± 5). OVX, ovariectomy; 3xTg-AD, triple transgenic mouse model of Alzheimer’s disorder. Data are shown as mean ± SEM, *p < 0.05, **p < 0.01, ***p < 0.001, ****p < 0.0001.

Body composition (body weight, fat, lean, free water, and total water) measurements were performed with a body composition analyzer for live small animals (EchoMRI™-700, EchoMRI LLC, Houston, TX), as described by the manufacturer. The animals were put in a restrainer and placed in the MRI machine for approximately 1 min (**Figure 2A**). The measurement was done in duplicate consecutively, without a time gap, and averaged. The body fat and lean weight were expressed as percentage of the body weight, and hydration ratio was calculated as the following:


$$HR=\frac{total\;water-free\;water}{lean}\times 100$$

### 2.4 Behavioral tests

#### 2.4.1 Cognitive behavioral tests

##### 2.4.1.1 Y-maze test

**Figure 3:**
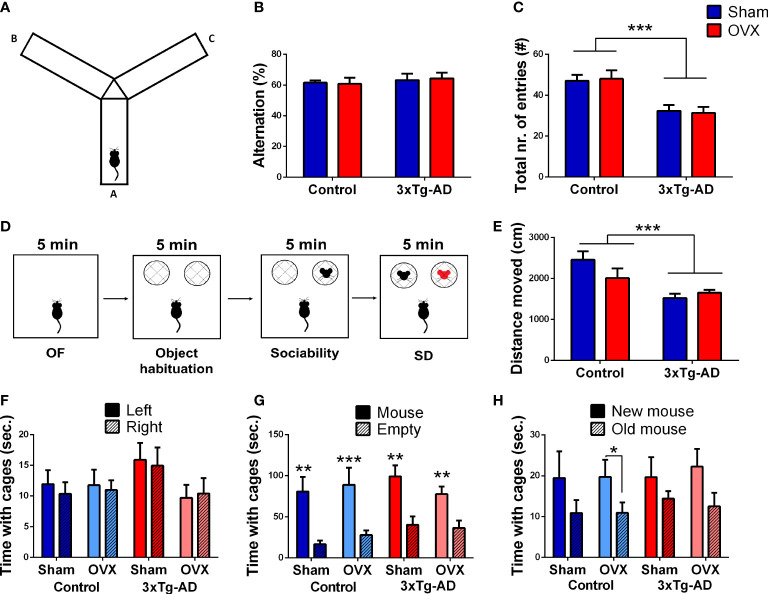
Y-maze and social discrimination (SD) tests. **(A)** Representative image of the Y-maze apparatus with three arms (A, B, and C). **(B)** Percentage of the good alternation in the Y-maze test. There was no significant difference between the groups. **(C)** Locomotor activity of the animals represented by the total number of entries. 3xTg-AD animals moved less than controls (*p* = 0.0001), without any OVX effect. **(D)** Representative figure of the different phases of the SD test. **(E)** Open-field (OF) test was the first 5-min phase of the SD test. A decreased locomotor activity, expressed in the distance moved (cm), was seen in the 3xTg-AD groups (*p* = 0.0008). **(F)** Object habituation phase of the SD test. No side preference was detected; thus, the next phases did not require any correction. **(G)** Sociability phase of the SD test. Every test mouse showed more interest towards the stimulus mouse containing cage (*p *< 0.0001); asterisks (*) show the result from the single-sample *t*-test against 50%. **(H)** SD phase. An increased interest towards the new mouse was detected in all groups (*p* = 0.0071). OVX, ovariectomy; 3xTg-AD, triple transgenic mouse model of Alzheimer’s disorder. Data are shown as mean ± SEM, **p < 0.01, ***p < 0.001, *p < 0,05.

The test was performed in a Y-shaped apparatus, with 3 arms (A, B, and C), with 30 × 7 × 20 cm dimensions, and in a 15–20 lux environment (**Figure 3A**) (57). Mice were placed in arm A and were allowed to explore the maze freely for 10 min. Before the entry of each animal, the maze was cleaned with 70% ethanol. Locomotion was calculated based on the total number of entries, while the spontaneous alternation reflects short-term memory and was calculated as the percentage (%) of “correct” alternation/total alterations. “Correct” alternation means entry into all three arms on consecutive choices (i.e., ABC, BCA, or CAB). Parameters were measured manually by an experimenter blind to the treatment groups.

##### 2.4.1.2 Social discrimination test

The test was performed in a 40 × 40 × 15 cm apparatus under red light (69). The experiment consisted of four phases, each lasting 5 min (**Figure 3D**). Firstly, the mice were placed in the box for acclimatization [open-field phase (OF)]. Secondly, two metal cages were placed into the box and fear from objects as well as side preference was evaluated. The goal was to habituate the animals to the container (object habituation). Then, a stimulus mouse [C57BL6, 25- to 30-day-old male, test naïve, sexually immature (70)] was placed under one of the metal cages (sociability phase). In the last 5 min, another stimulus mouse was placed under the other metal cage, and the position of the two cages was swapped [social discrimination (SD)]. The mice were left to explore freely the two animals. In the OF, the distance moved, and the time spent in the central or peripheral zone was analyzed automatically by EthoVision XT (Noldus IT, Wageningen, The Netherlands, version 15). Other parts of the test were analyzed using Solomon Coder (Solomon Coder, Hungary; https://solomoncoder.com/) by an experimenter blind to the treatment groups. The time and frequency sniffing the left or right container were evaluated. The sociability index (third phase) was calculated as:


$$SI=\frac{time\;spent\;sniffing\;the\;mice\;container}{time\;spent\;sniffing\;the\;mice\;container+empty\;container}*100$$

The discrimination index (DI, fourth phase) was calculated as:


$$DI=\frac{time\;spent\;sniffing\;new-old\;mice}{time\;spent\;sniffing\;new+old\;mice}$$

##### 2.4.1.3 Morris water maze test

**Figure 4:**
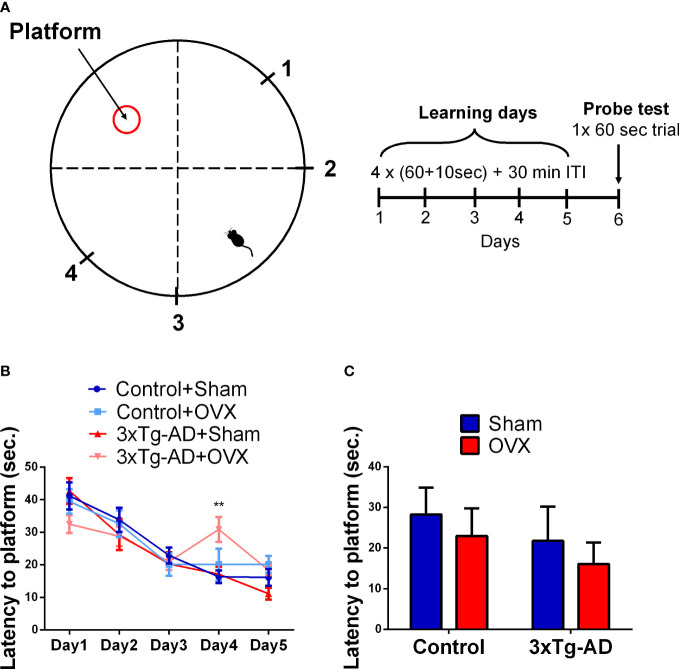
Morris water maze (MWM) test. **(A)** Representative figure of the MWM circular pool, with the location of the hidden platform, and the four starting points marked with 1–4. The learning phase consisted of 5 days; on each day, 4 × (60 + 10)-s trials were performed, with 30-min intertrial intervals (ITI). In the probe day (6th) one 60-s trial was done without the platform. **(B)** Latency to platform in seconds during the 5-day learning period. An improvement during the learning phase was seen in all groups (*p *< 0.0001). An interaction between OVX and time was detected, with a flatter learning curve of the OVX groups (*p* = 0.0004). **(C)** Latency to reach the platform on the probe day. No significant difference was found between the groups in the spatial memory. OVX, ovariectomy; 3xTg-AD, triple transgenic mouse model of Alzheimer’s disorder. Data are shown as mean ± SEM. **p<0,01.

A plastic circular pool (90 cm in diameter and 40 cm in height) was filled with tap water (24 ± 2°C), made opaque by white wall paint (**Figure 4A**) (38). A platform (6 cm in diameter) was placed 1 cm above the water for learning day 1, then moved 1 cm lower than the level of the water for days 2–5. The apparatus was divided into four quadrants and the platform was installed in the middle of one quadrant. Mice were released into the water from different points across trials (**Figure 4A**, marks 1–4) and were allowed to swim freely for 60 s to find the platform. If the mice could not find the platform during the 1 min, then it was guided there and left on the platform for 10 s. The learning phase (days 1–5) consisted of four trials with 30-min intertrial interval (ITI) when the animals were dried by a towel and returned to their home cages. On day 6 (probe day), the platform was removed from the water and the mice had 60 s to search for it. Latency to reach the platform during the learning phase was recorded manually, while during the probe test, time spent in different zones was calculated by EthoVision XT 15.

##### 2.4.1.4 Operant conditioning test

**Figure 5:**
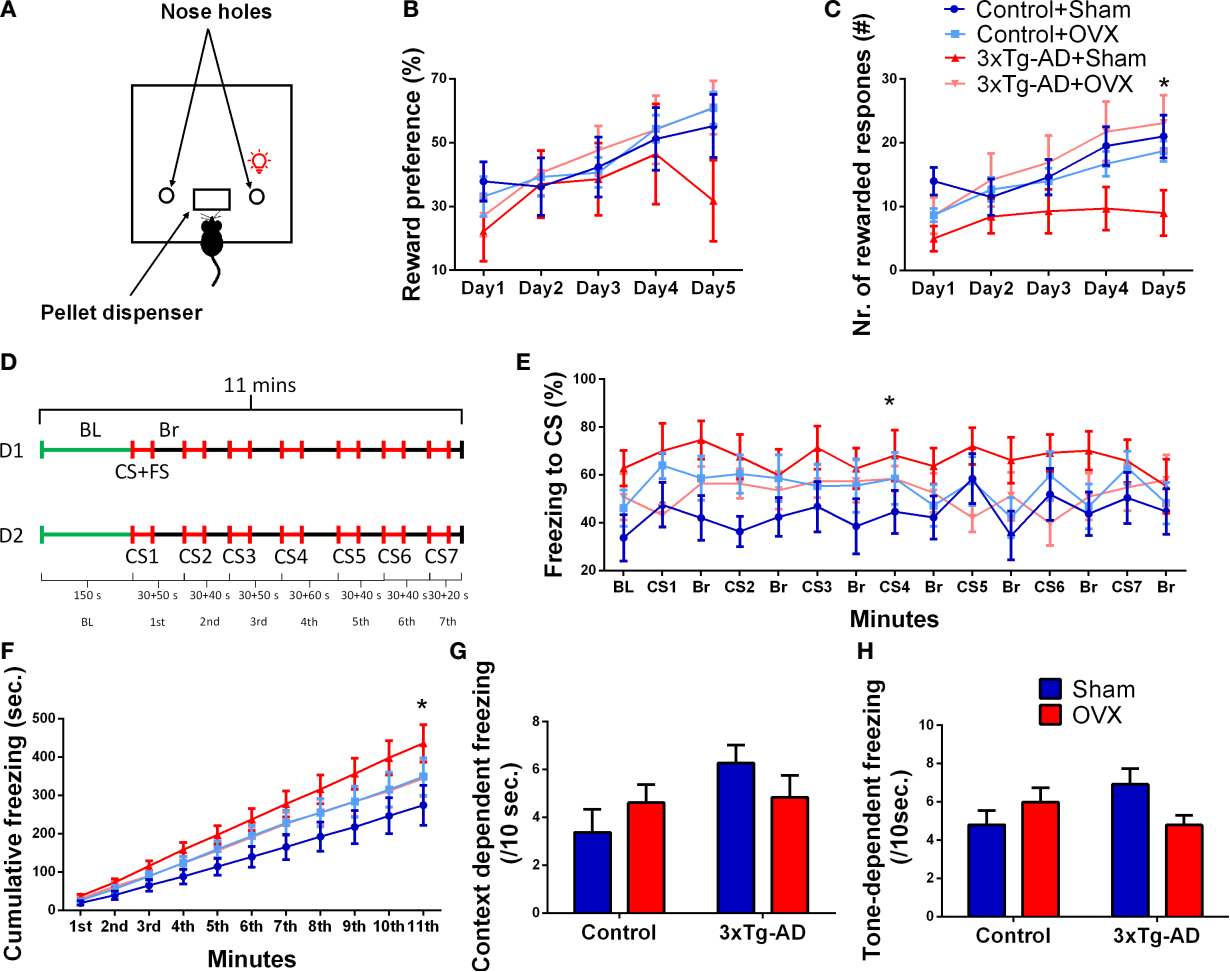
Operant conditioning (OC) and conditioned fear (CFT) tests. **(A)** Representative figure of the OC apparatus, with the light on above the reward associated nose hole. **(B)** Reward preference (ratio of responses on the rewarded vs. all nose pokes) during the OC test. An improvement during time was seen in all groups (*p *< 0.0001). **(C)** Number of rewarded responses during the OC test. Besides the time effect (*p *< 0.0001), a tendency for genotype × OVX interaction was also detected (*p* = 0.0707). 3xTg-AD animals after OXV responded significantly more than the Sham-operated ones (*p* = 0.0407). **(D)** Representative timeline of the 2 days (D1 and D2) lasting CFT test. D1 started with a 2.5-s baseline (BL) measurement, followed by a 30-s-long conditioned stimulus (CS: 80 dB pure tone at 7 kHz), which was co-terminated with an unconditioned stimulus [foot shock (FS): 0.7 mA, 1 s long, seven times in total], for a total of 11 min with random intertrial intervals (ITI, or break, Br). On D2, the experiment was repeated except that the animals did not receive an FS at the end of the CS. **(E)** Time spent freezing during CS and Br periods. For comparability, the values were calculated to 10-s bins. The AD × OVX interaction on CS meant that in the Control-Sham group, OVX aggravated, while in the 3xTg-AD group, the immobility was diminished (*p* = 0.0375). **(F)** The cumulative time spent in freezing (in 1-min bins) showed interaction between genotype, OVX, and time (*p* = 0.0005) with similar differences as seen on subgraph **(E)**. **(G)** Context and **(H)** tone or CS-dependent freezing (/10 s) during CFT. Repeated-measures ANOVA on time showed a significant elevation in freezing after CS (*p* = 0.0075). Again, a tendency for genotype and OVX interaction was detected (*p* = 0.0531), mainly due to the differences during tone dependency (*p* = 0.0251). OVX, ovariectomy; 3xTg-AD, triple transgenic mouse model of Alzheimer’s disorder. Data are shown as mean ± SEM, *p < 0.05.

The test was performed in an automated operant chamber (Med Associates, St. Albans, VT, USA) with two nose holes (**Figure 5A**) (57). As a reward, 45 mg of food pellets (Bio-Serv Dustless Precision Rodent Pellet, Bilaney Consultants GmbH, Germany) was used (71). Animals were placed inside a test chamber for 30 min to freely explore the environment. A nose poke into one of the nose holes was immediately associated with a reward followed by a 25-s-long time out with the chamber light switched on (time-out period), while the other nose hole was not baited (incorrect). During the time-out period, responses were not rewarded, but were registered. The test was divided into two phases: habituation (days 1–2) and learning (days 3–7), and data only from the learning phase is shown (**Figures 5B, C**, days 1–5). Reward preference (RP) (ratio of responses on the rewarded nose hole) was calculated. Number of rewarded responses and time-out reward hole nose pokes were also recorded.


$$RP=\;\frac{correct\;nose\;poke}{incorrect+correct\;nose\;poke}\;\times \;100$$

##### 2.4.1.5 Conditioned fear test

The mouse was placed into a Plexiglas chamber (25 × 25 × 30 cm) with an electrical grid floor (Coulbourn Instruments) that delivered the foot shocks (SuperTech Instruments). For 2.5 min, the animals were left in the boxes for habituation [baseline (BL)]. Then, at pseudorandom intervals (60–90 s), a 30-s-long conditioned stimulus (CS: 80 dB pure tone at 7 kHz) was played and co-terminated with an unconditioned stimulus (foot shock: 0.7 mA, 1 s long, seven times in total), for a total of 11 min (**Figure 5D**). The following day, the experiment was repeated, except that the animals did not receive foot shocks at the end of the CSs (72). The chambers were cleaned with soap water and water after every trial. The experiment was conducted in bright light (700 lux). Time spent in immobility was measured automatically by Ethovision XT 15 on the second day. Time spent in immobility was calculated for the BL (mean for 10 s) as well as for CSs (mean for 7 CS per 10 s).

#### 2.4.2 Anxiety-related behavioral tests

##### 2.4.2.1 Elevated plus maze test

**Figure 6:**
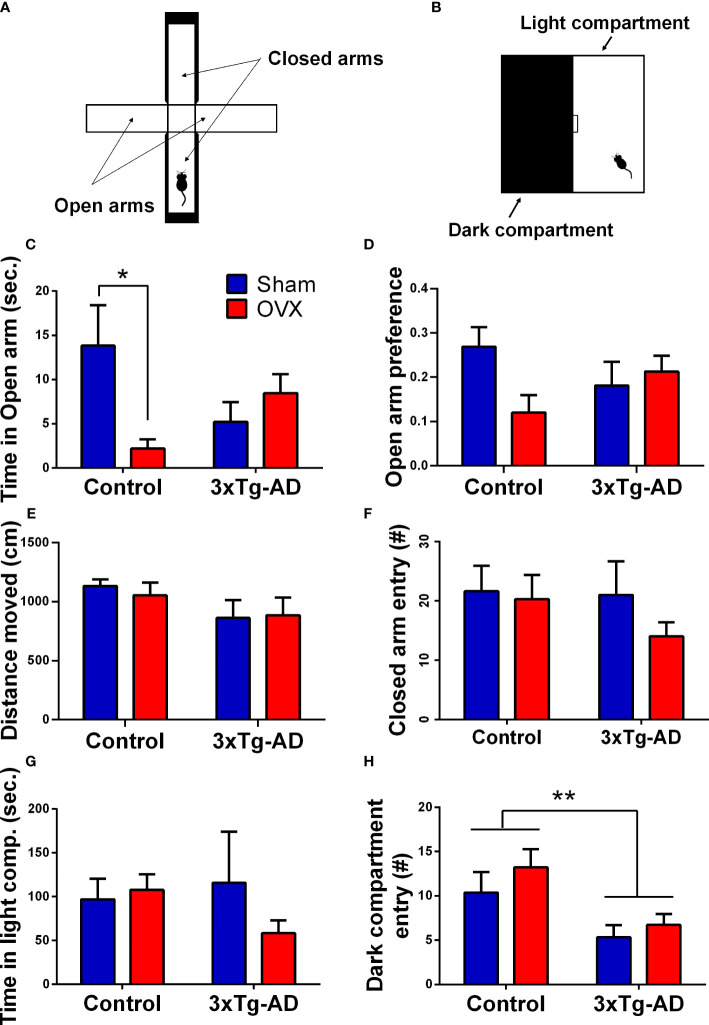
Elevated plus maze (EPM) and light–dark box (LD) tests. **(A)** Representative figure of the EPM apparatus, with two open and two closed arms. **(B)** Representative image of the LD equipment, with a light and a dark compartment, separated with a small gateway. **(C)** Time spent in the open arm of the EPM. A significant interaction between genotype and surgery groups was detected (*p* = 0.0088). Control-OVX animals spent less time in the open arm compared to Control-Sham ones (*p* = 0.0192). **(D)** Open arm preference in the EPM test. A significant interaction between genotype and surgery groups was seen (*p* = 0.0421). **(E)** Distance moved (cm) during the 5-min EPM test. No difference between the groups was detected. **(F)** Total number of entries into the closed arms in the EPM test. Differences between the groups were not significant. **(G)** Time spent in the light compartment during the LD test. No difference regarding genotype or OVX surgery was detected. **(H)** Number of entries in the dark compartment during LD test. 3xTg-AD mice moved significantly less, than controls (*p* = 0.0044), without any OVX effect. OVX, ovariectomy; 3xTg-AD, triple transgenic mouse model of Alzheimer’s disorder. Data are shown as mean ± SEM, *p < 0.05, **p < 0.01.

A plus-shaped device was used, which comprised two opposite open arms and two enclosed arms (30 × 7 × 30 cm) (**Figure 6A**) (73). The mice were placed in the center of the apparatus facing the open arm and were allowed to explore the maze for 5 min. Before the entry of each animal, the maze was cleaned with 70% ethanol. The time spent and number of entries into the different arms as well as the distance moved (cm) were quantified with EthoVision XT 15. The open arm preference (OP) (74) was calculated as:


$$OP=\;\frac{open\;arm\;entries}{open\;arm\;entries+closed\;arm\;entries}$$

##### 2.4.2.2 Light–dark box test

LD was performed in a 40 × 20 × 25 cm box, which had two compartments: a light (white colored) compartment that is open from above and a dark (black colored) compartment that is closed from every side (**Figure 6B**). A small gate (5 × 5 cm) between the two compartments, where the animal can freely pass, was present. The mice were placed in the light part of the box and were allowed to explore the environment for 10 min. The duration of time spent in each compartment, the total number of entries, and latency to dark compartments were measured by Solomon Coder.

##### 2.4.2.3 Fox odor avoidance test

**Figure 7:**
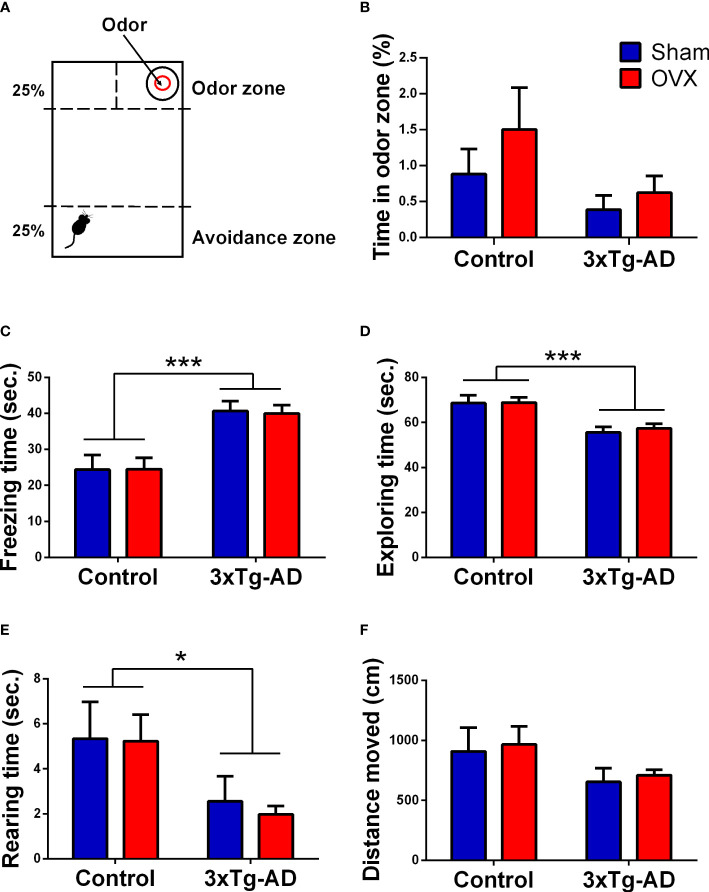
Fox odor test (FOT). **(A)** Representative figure of the FOT apparatus, presented with the odor zone [where the 2-methyl-2-thriazoline (2MT, fox odor) was placed] and the avoidance zone (distant part from the odor). **(B)** Time (in seconds) spent in the odor zone. No significant difference between genotypes or between surgery groups was detected. **(C)** Time (in seconds) spent freezing. 3xTg-AD mice spent more time freezing compared to control groups (*p *< 0.0001). **(D)** Time (in seconds) spent exploring the FOT box. The 3xTg-AD group showed reduced exploration time compared to controls (*p *< 0.0001). **(E)** Time (in seconds) spent rearing. A genotype effect was visible with less vertical movement in 3xTg-AD animals (*p* = 0.0118). **(F)** Distance moved (cm). A tendency for genotype difference was seen, with the 3xTg-AD mice moving less than controls (*p* = 0.0723). OVX, ovariectomy; 3xTg-AD, triple transgenic mouse model of Alzheimer’s disorder. Data are shown as mean ± SEM, *p < 0.05, ***p < 0.001.

Exposure to fox-derived synthetic predator odor, 2-methyl-2-thriazoline (2MT, #M83406, Sigma Aldrich), was performed in a separate experimental room under a fume hood. A transparent Plexiglas arena (43 × 27 × 19 cm) was used (**Figure 7A**). During the test, a 2MT solution-soaked filter paper (40 μl in 1 ml of distilled water, 50 μl/animal) was placed in a plastic 50-ml conical tube cap in one corner of the box (75). A 7 × 11 cm “odor zone” around the odor source was defined. The opposite part (25%) of the box was appointed as “avoidance zone”. During the test, the animal was placed in the avoidance zone and left to freely explore the arena for 10 min. Time spent in the odor zone and the distance moved (cm) was measured with EthoVision XT 15. Different anxiety-related behaviors, like the time spent freezing, grooming, and sniffing as well as the exploratory behaviors like time spent rearing and exploring was analyzed manually by Solomon Coder by an experimenter blind to the treatment groups.

### 2.5 Histological evaluations

#### 2.5.1 Hematoxylin–eosin staining for uterus morphology

**Figure 8:**
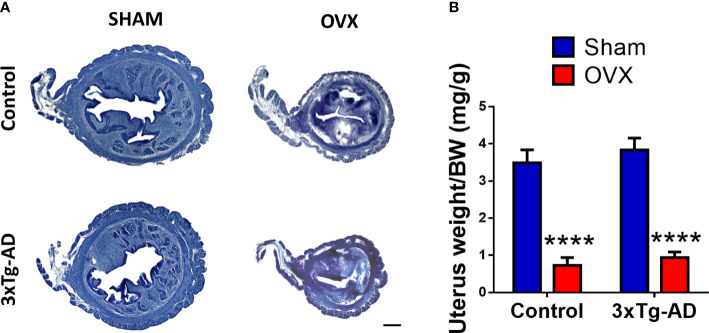
Changes in the uterus 2 months after Sham or OVX surgery. **(A)** Representative figure of the uterus stained with hematoxylin–eosin (HE). A decrease in size and epithelial layer thickness, and damaged integrity of the endometrial glands is visible. **(B)** Uterus weight normalized to the body weight (BW) of the animals. A significant decrease was seen after OVX surgery (*p *< 0.0001). OVX, ovariectomy; 3xTg-AD, triple transgenic mouse model of Alzheimer’s disorder. Data are shown as mean ± SEM, ****p < 0.0001. Scale bar, 200 µm.

After weighing, uteruses were fixed in 4% PFA for 24 h, then dehydrated with 30% sucrose solution. Thirty-micrometer slices were made with a freezing microtome (Leica SM2010 R). Hematoxylin–eosin (HE) staining was performed on the slices to see morphological changes in the epithelium layer thickness, lumen size, and the integrity of the endometrial glands (**Figure 8A**). Samples were imaged with a Nikon Eclipse E1 R (Nikon, Tokyo, Japan) microscope at 4× magnification.

#### 2.5.2 Amyloid-β_1-42_ and choline acetyltransferase immunohistochemistry

For Aβ_1-42_ and ChAT staining, peroxidase-based immunohistochemistry with nickel-diaminobenzidine tetrahydrochloride (Ni-DAB) visualization was undertaken (17). Firstly, only for the Aβ staining, a 10-min concentrated formic acid (Sigma-Aldrich, #F0507) exploration was implemented. Secondly, endogen peroxidase was blocked by a 3% peroxide (H_2_O_2_) solution. After blocking, slices were incubated 72 h with the primary antibody recognizing Aβ (Rabbit, 1:500, Invitrogen, #71-5800) or ChAT (Goat, 1:1,000, Millipore, #AB144P). After 72 h, brain slices were incubated with a biotinylated secondary antibody (biotinylated anti-rabbit, 1:200, Jackson ImmunoResearch, #111-065-003 or biotinylated anti-goat 1:200, Jackson ImmunoResearch, #705-065-147) at room temperature (RT), for 2 h. An avidin–biotin kit (VECTASTAIN Elite ABC-Peroxidase Kits, PK-6100, Vector Laboratories) was used for 2 h, RT, to detect biotinylated molecules. Then, the visualization was performed with a Ni-DAB and glucose oxidase mixture. Samples were imaged with a Nikon Eclipse E1 R (Nikon, Tokyo, Japan) microscope at 4× magnification.

**Figure 9:**
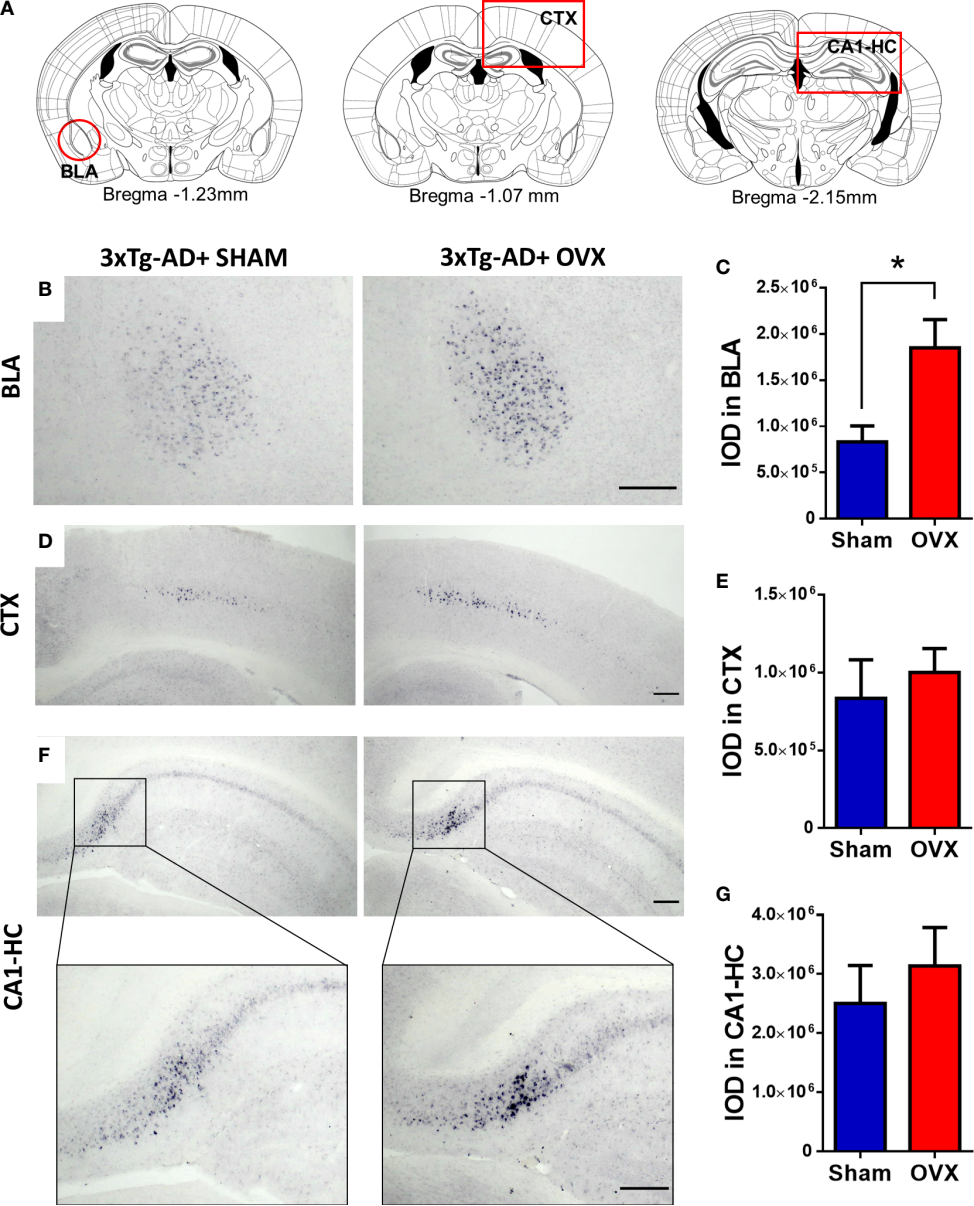
Immunohistochemical staining (NiDAB) of amyloid-β_1-42_ (Aβ) plaques in different brain regions. There was no Aβ signal detectable in the brain of control animals; therefore, we compared 3xTg-AD with and without ovariectomy (OVX). **(A)** Representative figures based on the Paxinos Mouse Brain atlas (4th Edition) about the brain regions of interest, framed with red: Basolateral amygdala (BLA), at Bregma −1.23 mm, Motor and somatosensory cortex (CTX) at Bregma −1.07 mm, and CA1 hippocampal region (CA1-HC) presented at Bregma −2.15 mm. **(B)** Representative pictures of Aβ plaques in the BLA of the 3xTg-AD animals after Sham or OVX surgery. **(C)** The integrated optical density (IOD) of Aβ plaques measured in the BLA. A significant increase was detected after OVX surgery (*p* = 0.0236). **(D)** Representative pictures of Aβ plaques in the CTX in 3xTg-AD animals after Sham or OVX surgery. **(E)** The IOD of Aβ plaques measured in the CTX. No significant difference was detected. **(F)** Representative pictures of Aβ plaques in the CA1-HC of 3xTg-AD animals after Sham or OVX surgery, with a close-up to a small part of the CA1 region. **(G)** The IOD of Aβ plaques measured in the HC. The difference between the two surgery groups was not significant. 3xTg-AD, triple transgenic mouse model of Alzheimer’s disorder. Data are shown as mean ± SEM, *p < 0.05. Scale bar: 200 µm.

**Figure 10:**
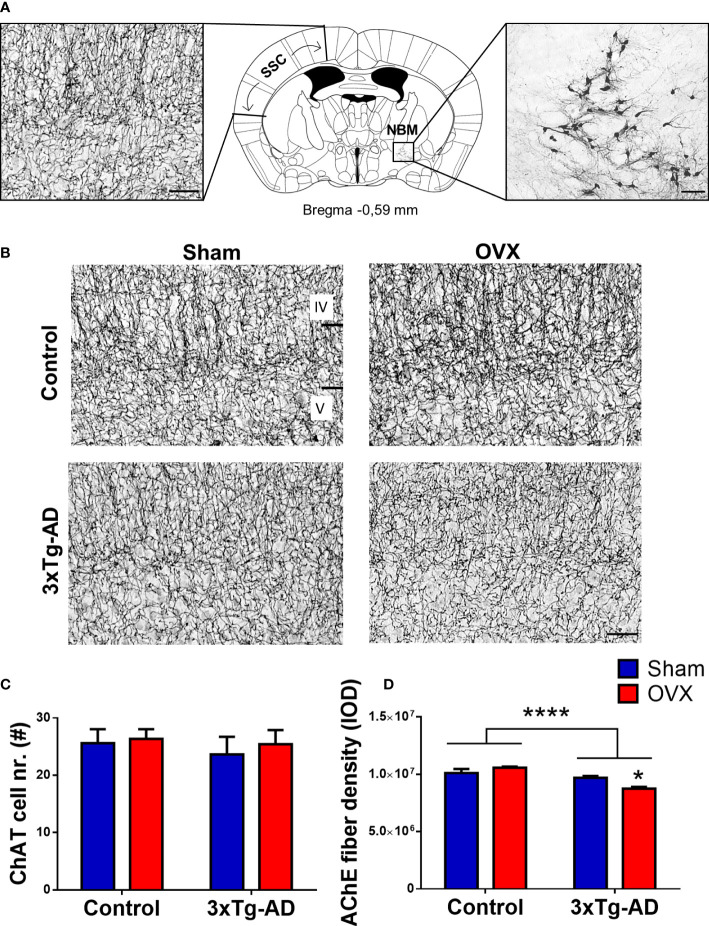
Immunohistochemical and histochemical staining of the cholinergic cell bodies and fibers. **(A)** Neuroanatomical location of the cholinergic choline-acetyltransferase (ChAT)-positive neurons in the nucleus basalis magnocellularis (NBM) and their acetylcholinesterase (AChE)-positive fibers in the somatosensory cortex (SSC). Schematic coronal brain section was adapted from Franklin and Paxinos (4th Edition) Mouse Brain atlas. **(B)** Representative pictures of the AChE-positive fibers in layers IV and V of SSC. Black bars indicate layers IV and V of the SSC. **(C)** Number of ChAT-positive cell bodies in the NBM region, stained with NiDAB immunohistochemistry. No significant difference was detected between groups. **(D)** AChE-positive fiber density measured in the SSC, expressed in integrated optical density (IOD). 3xTg-AD mice have a lower AChE fiber density compared to controls (*p *< 0.0001), with a significant interaction between groups (*p* = 0.0025). The decrease in density was exacerbated by OVX surgery in the 3xTg-AD group (*p* = 0.0147). OVX, ovariectomy; 3xTg-AD, triple transgenic mouse model of Alzheimer’s disorder. Data are shown as mean ± SEM, *p < 0.05, ***p < 0.001. Scale bar: **(A)** AChE staining in the SSC, 50 µm, and ChAT staining in the NBM, 100 µm. **(B)** 50 µm.

In case of Aβ plaques, the integrated optical density (IOD) was measured by ImageJ/Fiji in the basolateral amygdala (BLA), the somatosensory and motor cortex (CTX) between Bregma 0.50 mm and −1.20 mm, and the CA1 region of the hippocampus (CA1-HC) between Bregma −1.19 mm and −2.69 mm (**Figure 9A**). In other brain areas of 5-month-old 3xTg-AD mice, no amyloid deposition was found. After ChAT staining, the number of ChAT-positive cells was counted in the NBM, a brain region containing cholinergic cell bodies, and highly affected in AD (**Figure 10A**) (76, 77).

#### 2.5.3 Acetylcholinesterase histochemistry

To label cholinergic fibers in the somatosensory cortex (SSC), the target area of the NBM neurons (78), an AChE histochemistry was performed (17). Slices were selected from the coordinates: Bregma +0.50 mm to −1.06 mm (**Figure 10A**). Free-floating brain slices were incubated in a mixture of sodium acetate buffer (0.1 M; pH 6) acetylthiocholine iodide (0.05%), sodium citrate (0.1 M), copper sulfate (0.03 M), and potassium ferricyanide (5 mM). This was followed by ammonium sulfide (1%) and then silver nitrate (1%) incubation (17, 79). Analysis was performed with ImageJ/Fiji software. Samples were imaged with a Nikon Eclipse E1 R (Nikon, Tokyo, Japan) microscope at 10× magnification. IOD was measured between layer IV and V of the SSC (**Figure 10A**).

### 2.6 Z-score calculations

Integrated z-score was calculated for four major parameters: somatic, cognitive, anxiety, and locomotion, as proposed by Guilloux et al. (80), and previously presented in (73, 81). For each parameter, a normalized value (studentization) was calculated according to the following equation:


$$z-score=\;\frac{individual\;value-mean_{control}}{standard\;deviation_{control}}$$

and the included parameters were adjusted to have the same directionality. Somatic z-score was calculated from the averages of body weight change, fat/BW percentage, and uterus weight (×−1) z-scores. Cognitive z-score was calculated from alteration in the Y-maze; the area under the curve (AUC) of the latencies to platform during learning days 1–5 in MWM (×−1), and latency to platform on the probe day (×−1) in MWM; average freezing during baseline and conditioned stimuli in CFT; and the AUC of the reward preference learning days 3–7 in operant conditioning. Anxiety z-score was averaged from the z-scores of open arm duration (×0.5) and open arm preference (×0.5) in EPM; time spent in light compartment in LD; time spent freezing (×−1) and percentage of time spent in the odor zone in FOT; and percentage of time spent with freezing in CFT day 2 (×−1). Locomotion z-score was calculated from the parameters that reflected mobility in the given experiment [distance moved in EPM (×0.5), OF and fox odor tests; total number of entries in the Y-maze, EPM (×0.5), and LD], then averaged for each animal. Somatic, cognitive, anxiety, and locomotor z-scores were averaged for every group and statistically tested. If multiple parameters indicating the same meaning within an experiment were included in averaged z-score calculations (e.g., distance moved and closed arm entries on EPM in the locomotion z-score), then they were multiplied by ×0.5 in order to avoid unwanted weighting of the specific test.

### 2.7 Statistical analysis

GraphPad Prism (version 6.0) was used for statistical analyses. Two-way ANOVA (MRI, Y-maze, OF, Sociability, SD, EPM, LD box, FOT, and histology; on factors genotype and OVX) or repeated-measures ANOVA (MWM, body weight change, OC, and CFT; additional factor: time) was used to compare the groups, followed by Tukey HSD or Sidak *post-hoc* test. For comparison of two groups, Student’s *t*-test was used (Aβ staining). All data are presented as mean ± SEM and *p*< 0.05 was considered as a statistically significant difference.

## 3 Results

### 3.1 Changes in body composition measured with MRI

Regarding body weight changes, a difference was found between the two genotypes [*F*_(3,59)_ = 12.59, *p*< 0.0001], the 3xTg-AD mice being heavier than the controls. However, OVX surgery itself increased the body weight during a 40-day period [*F*_(1,19)_ = 16.35, *p* = 0.0007] without any influence of the genotype (**Figure 2B**). This increased body weight can be explained by the increased body fat ratio, where a genotype effect was also detectable with more fat in 3xTg-AD animals [*F*_(1,32)_ = 10.01, *p* = 0.0034] (**Figure 2C**). OVX was able to increase the fat accumulation in both genotypes [*F*_(1,32)_ = 38.38, *p*< 0.0001] (**Figure 2C**). Simultaneously, lean body weight ratio decreased [genotype: *F*_(1,32)_ = 11.97, *p* = 0.0016, OVX: *F*_(1,32)_ = 47.45, *p*< 0.0001] (**Figure 2D**). A significant negative correlation between body fat and lean ratio was also detected (*r* = −0.9870, *p*< 0.0001) (**Figure 2E**). The hydration ratio (((total water − free water)/lean)*100) of all animals was in the normal range (80 ± 5%), without any effect of genotype or OVX (**Figure 2F**).

### 3.2 Behavioral tests

#### 3.2.1 Cognitive behavioral tests

##### 3.2.1.1 Y-maze test

There was no difference between the groups in the main parameter of short-term memory, the alternation (**Figure 3B**). The 3xTg-AD mice moved significantly less compared to control animals [*F*_(1,31)_ = 19.52, *p* = 0.0001] without any effect or influence of OVX (**Figure 3C**).

##### 3.2.1.2 Social discrimination (SD) test

We confirmed the reduced locomotion of 3xTg-AD mice during the first 5 min OF phase [*F*_(1,32)_ = 13.80, *p* = 0.0008], without OVX effect (**Figure 3E**). Neither the genotype, nor the OVX influenced the number of entries or time spent in the centrum, not even if we corrected it with locomotion (**Table 1**).

**Table 1:** Detailed results of the social discrimination tests.

Test Phase	Behavior		Experimental groups								Statistics		
			Control		Control		3xTg-AD		3xTg-AD	
			Sham		OVX		Sham		OVX		*p*-value		
			Average	SEM	Average	SEM	Average	SEM	Average	SEM	Genotype	OVX	Interaction
**Openfield phase**	% time spent in centrum		17.01	2.98	16.60	3.33	16.55	4.71	20.22	4.31	0.6955	0.6865	0.6135
	Centrum frequency		32.00	3.59	28.40	3.65	25.29	5.12	24.91	3.06	0.1901	0.6055	0.6752
**Habituation phase**	Sniffing frequency	Cage inside	20.38	2.57	19.30	3.59	25.86	4.54	13.45	2.79	0.9099	0.0817	0.1474
		Cage outside	16.13	2.36	16.30	2.65	21.14	5.18	13.27	2.86	0.6118	0.3327	0.3072
	∑	36.50	3.75	35.60	5.67	47.00	9.14	26.73	5.26	0.7353	0.1298	0.1696
	% time spent with sniffing cages		22.29	3.18	22.74	3.34	30.86	5.16	20.12	4.28	0.3331	0.3076	0.2595
	Bout length		0.58	0.06	0.69	0.08	0.66	0.11	0.67	0.08	0.5417	0.3646	0.7367
**Sociability**	Sniffing frequency	Cage with mouse	18.38	4.16	13.90	2.05	22.14	2.09	15.64	2.40	0.1960	0.0881	0.9309
		Empty cage	7.25	1.91	13.80	2.19	14.29	1.80	11.18	1.93	0.1979	0.2901	0.0391
	SI		76.40	6.18	72.97	4.18	71.52	6.14	70.16	5.17	0.4810	0.6602	0.8499
	Bout length mouse		3.73	0.52	4.85	0.88	6.57	1.88	5.20	0.73	0.5915	0.5915	0.4337
**Social discrimination**	Sniffing frequency	Known mouse	16.38	3.19	16.30	2.88	14.86	1.52	14.00	2.73	0.5045	0.8701	0.8909
		Unknown mouse	16.25	1.41	18.00	1.80	14.57	2.95	14.82	2.74	0.3173	0.6792	0.7554
	DI		0.20	0.17	0.25	0.10	0.06	0.11	0.30	0.15	0.7194	0.2943	0.4958
	Bout length two mice		2.82	0.63	2.86	0.56	3.43	0.53	3.24	0.45	0.3772	0.8899	0.8411

Data are expressed as mean ± SEM. The results of the statistical analysis (two-way ANOVA) are presented. Significant differences are marked with red, bold numbers. OVX, ovariectomy; 3xTg-AD, triple transgenic mouse model of Alzheimer’s disorder, SI, Sociability index, DI, Discrimination index.

During the object habituation phase, none of the mice preferred any side; thus, the next phases did not require any correction (**Figure 3F**). OVX did not significantly affect the number of object approaches as well [*F*_(1,32)_ = 3.05, *p* = 0.1298; **Table 1**].

In the sociability phase, every mouse showed more interest to the stimulus mouse-containing cage [repeated-measures ANOVA: *F*_(1,32)_ = 33.81, *p*< 0.0001; single-sample *t*-test against 50%; Control-Sham: *t*_(7)_ = 4.27, *p* = 0.0037; Control-OVX: *t*_(9)_ = 5.49, *p* = 0.0004; 3xTg-AD-Sham: *t*_(6)_ = 3.50, *p* = 0.0128; 3xTg-AD-OVX: *t*_(9)_ = 3.90, *p* = 0.0036] without significant difference between groups (**Figure 3G**). There was a tendency for OVX animals to approach the social container a fewer number of times [*F*_(1,32)_ = 3.89, *p* = 0.0881; **Table 1**].

In the social discrimination phase, an increased interest towards the new mouse was detected in all groups [*F*_(1,62)_ = 7.75, *p* = 0.0071], suggesting that—in general—the test animals preferred the new stimulus mice, as expected (**Figure 3H**). However, when we checked the groups one by one, only the Control-OVX group seemed to have intact memory with a tendency in the 3xTg-AD-OVX group [single-sample *t*-test against 0; Control-Sham: *t*_(7)_ = 1.17, *p* = 0.2806; Control-OVX: *t*_(9)_ = 2.48, *p* = 0.0348; 3xTg-AD-Sham: *t*_(6)_ = 0.53, *p* = 0.6179; 3xTg-AD-OVX: *t*_(9)_ = 2.03, *p* = 0.0732]. A larger number of animals are probably needed for this test to work properly. Nevertheless, there was no overall difference between groups.

##### 3.2.1.3 Morris water maze test

The latencies to reach the platform showed a significant improvement in time during the learning phase, independently from genotypes or surgery [*F*_(4,132)_ = 43.42, *p*< 0.0001] (**Figure 4B**). At the end of the 5th day, the animals were able to find the platform within 20 s (o average: 17.01 ± 1.21 s), suggesting that all groups learned the task. A significant interaction between OVX and time was detected [*F*_(4,128)_ = 4.31, *p* = 0.0026]; the OVX groups started to learn the task a bit later, as day 2 was not significantly different from day 1 in contrast to Sham-operated groups. Moreover, during days 4 and 5, the OVX groups differed significantly from the Sham-operated ones [*F*_(1,32)_ = 6.09, *p* = 0.0191], suggesting a flatter learning curve. Additionally, the fluctuation observable in the 3xTg-AD-OVX group suggests random choice, thus, not appropriate learning of this group. The genotype also showed a tendency for time dependence [*F*_(4,128)_ = 2.18, *p* = 0.0744], with a subtle learning impairment of the 3xTg-AD mice. All the animals remembered the place of the platform as during the probe test they spent more than 25% of the time in the platform quadrant [single-sample *t*-test against 25; Control-Sham: *t*_(7)_ = 2.14, *p* = 0.0701; Control-OVX: *t*_(9)_ = 5.50, *p* = 0.0004; 3xTg-AD-Sham: *t*_(6)_ = 6.12, *p* = 0.0009; 3xTg-AD-OVX: *t*_(9)_ = 3.02, *p* = 0.0130]. No difference was found between groups in the probe day in the latency to reach the platform or time spent in the quadrant, where the platform was during the probe day (**Figure 4C**).

##### 3.2.1.4 Operant conditioning test

In reward preference, an improvement during time was detected [*F*_(4,128)_ = 9.73, *p*< 0.0001] without any influence of the genotype or surgical removal of the ovaries (**Figure 5B**). In the number of rewarded responses, a similar time effect was seen [*F*_(4,128)_ = 16.10, *p*< 0.001] (**Figure 5C**), with a tendency for genotype × OVX interaction [*F*_(1,32)_ = 53.49, *p* = 0.0707]. There was a tendency for 3xTg-AD-Sham-operated animals to respond fewer times than Control-Sham-operated ones (*p* = 0.0794), while 3xTg-AD animals after OXV responded significantly more than the Sham-operated ones (*p* = 0.0407).

##### 3.2.1.5 Conditioned fear test

We expressed the time spent in immobile posture during different phases as the percentage of the time period to get comparable values [i.e., the 150-s BL period is hardly comparable to the 30-s CS periods or random breaks (Br)] (**Figure 5E**). When we were concentrating on CS-induced changes, there was a significant interaction between the genotype and OVX [repeated-measures ANOVA on the seven CS: *F*_(1,31)_ = 4.72, *p* = 0.0375]; the OXV increased freezing in control, but decreased in 3xTg-AD mice. The same effect was also seen in the cumulative time spent in freezing (in 1-min bins) (**Figure 5F**); not surprisingly this time, the interaction was significant between all three (genotype, OVX, and time) factors [*F*_(10,320)_ = 53.26, *p* = 0.0005]. The time spent with inactivity during the initial context-dependent phase (BL, 150 s) (**Figure 5G**) and during the seven CS (conditioned phase, **Figure 5H**) was also calculated. Using repeated-measures ANOVA on context and cue-induced freezing, the CS, as a cue, significantly elevated the immobility time [*F*_(1,32)_ = 8.16, *p* = 0.0075]. There was a tendency again for genotype and OVX interaction [*F*_(1,32)_ = 4.03, *p* = 0.0531]. This was due to the significant interaction during tone-dependent freezing [*F*_(1,32)_ = 5.53, *p* = 0.0251] (**Figure 5H**) as no difference was detectable in the context-dependent phase [*F*_(1,32)_ = 3.12, *p* = 0.0871] (**Figure 5G**).

#### 3.2.2 Anxiety-related behavioral tests

##### 3.2.2.1 Elevated plus maze test

There was a significant interaction between genotype and OVX in the time spent in open arms [*F*_(1,32)_ = 7.774, *p* = 0.0088], and in open arm preference [*F*_(1,32)_ = 4.484, *p* = 0.0421] (**Figures 6C, D**), but no difference was detected in the time spent in the closed arm [time %; Control-Sham: 239.52 ± 9.99, Control-OVX: 250.09 ± 10.68, 3xTg-AD-Sham: 237.62 ± 14.09, 3xTg-AD-OVX: 260.13 ± 7.06; genotype: *F*_(1,32)_ = 0.1554, *p* = 0.6961; OVX: *F*_(1,32)_ = 2.569, *p* = 0.1188]. More specifically, Control-OVX animals spent less time in the open arm compared to the Control-Sham group (*p* = 0.0192), whose effect was not detectable in 3xTg-AD mice. In the locomotion parameters, similar to distance moved and the number of entries into the closed arms, no significant differences were detected between the groups (**Figures 6E, F**).

##### 3.2.2.2 Light–dark box test

No differences were seen in the anxiety-related parameters like time spent in the light compartment (**Figure 6G**). However, in the locomotor activity represented by the number of entries to the dark compartment, a genotype effect was detected [*F*_(1,30)_ = 9.80, *p* = 0.0039] (**Figure 6H**). 3xTg-AD animals moved significantly less than the controls.

##### 3.2.2.3 Fox odor test

A tendency for decreased time spent in the odor zone was seen in the 3xTg-AD animals compared to the control groups [*F*_(1,27)_ = 3.51, *p* = 0.0719] (**Figure 7B**). Accordingly, the 3xTg-AD animals spent more time freezing [*F*_(1,31)_ = 25.33, *p*< 0.0001] (**Figure 7C**) and reared [*F*_(1,31)_ = 7.15, *p* = 0.0118] (**Figure 7E**) and vertically explored the environment [*F*_(1,31)_ = 22.48; *p*< 0.0001] (**Figure 7D**) less than controls. These may suggest that 3xTg-AD animals were more anxious in the presence of a predator odor. A tendency of genotype difference was also seen in the locomotor activity, expressed by the distance moved [*F*_(1,31)_ = 3.46, *p* = 0.0723] (**Figure 7F**). Other parameters (like grooming and sniffing) were not different between groups and thereby not shown. The OVX surgery had no significant effect on the parameters examined during FOT.

### 3.3 Z-scores

The somatic z-score showed a significant interaction between genotype and surgery groups [*F*_(1,32)_ = 41.35, *p* < 0.0001] (**Table 2**). Animals who underwent OVX surgery had a higher somatic z-score [*F*_(1,32)_ = 12.92, *p* = 0.0010], whose effect was more pronounced in Control than in 3xTg-AD mice. In cognitive z-score, no significant differences were detected between the groups. Anxiety z-score showed an interaction between genotype and surgery groups [*F*_(1,32)_ = 23.26, *p* < 0.0001]. Namely, OVX increased anxiety in Control, but decreased in 3xTg-AD animals. Indeed, in general, 3xTg-AD animals had a lower anxiety z-score, meaning more anxious behavior [*F*_(1,32)_ = 17.61, *p* = 0.0002]. The locomotor differences were also supported by its z-score. Namely, the 3xTg-AD animals had a lower z-score number, meaning, in general, they moved less [*F*_(1,32)_ = 19.64, *p* = 0.0001]. A significant interaction between genotype and surgery groups was also detected [*F*_(1,32)_ = 27.45, *p* < 0.0001]. More specifically, OVX reduced locomotion in Control, but not that much in 3xTg-AD mice, which was moving less even before that.

**Table 2:** Z-scores calculated from somatic, cognitive, anxiety, and locomotor parameters.

Type	Experimental groups	Z-score ± SEM	Genotype	Surgery	Interaction
**Somatic**	Control-Sham	(−0.0070) ± 0.1387	*p* = 0.2902	***p* = 0.0010**	***p* = 0.0000**
	Control-OVX	3.8905 ± 0.5631			
	3xTg-AD-Sham	0.6443 ± 0.4267			
	3xTg-AD-OVX	3.7049 ± 0.4227			
**Cognitiv**	Control-Sham	(−0.000) ± 0.1197	*p* = 0.6754	*p* = 0.3038	*p* = 0.2349
	Control-OVX	0.1442 ± 0.1645			
	3xTg-AD-Sham	0.0302 ± 0.2340			
	3xTg-AD-OVX	0.2686 ± 0.1861			
**Anxiety**	Control-Sham	0.0000 ± 0.3964	***p* = 0.0002**	*p* = 0.8743	***p* = 0.0000**
	Control-OVX	(−0.2518) ± 0.4862			
	3xTg-AD-Sham	(−2.0022) ± 0.2444			
	3xTg-AD-OVX	(−1.6222) ± 0.3366			
**Locomotor**	Control-Sham	0.000 ± 0.4750	***p* = 0.0001**	*p* = 0.6097	***p* = 0.0000**
	Control-OVX	(−0.4145) ± 0.6432			
	3xTg-AD-Sham	(−2.4225) ± 0.4375			
	3xTg-AD-OVX	(−2.5365) ± 0.3748			

Data are expressed as z-score (mean) ± SEM. Statistical data (two-way ANOVA) is presented. Significant differences are marked with red, bold numbers. OVX, ovariectomy; 3xTg-AD, triple transgenic mouse model of Alzheimer’s disorder.

### 3.4 Histological evaluations

#### 3.4.1 Uterus

The representative pictures with HE staining showed increased epithelium thickness, deteriorated endometrial glands, and a substantial difference between the size of the uterus and lumen (**Figure 8A**). Both in the control and 3xTg-AD groups, the normalized weight of the uterus was significantly lower after OVX compared to the Sham group [*F*_(1,31)_ = 121.80, *p*< 0.0001] (**Figure 8B**).

#### 3.4.2 Amyloid-β accumulation in different brain areas

Amyloid-β plaques were only quantified in 3xTg-AD mice, because no deposition was detected in the Control-Sham or Control-OVX groups (see **Supplementary Figure 1**). In the BLA, the 3xTg-AD-OVX mice had significantly more plaques than their Sham-operated mates [*t*_(9)_ = 2.72, *p* = 0.0236] (**Figures 9B, C**). In the CTX and CA1-HC, no significant effect of OVX was found (**Figures 9D–G**).

#### 3.4.3 Morphological changes in the cholinergic system

ChAT-positive cells were counted in the NBM region. We found no difference in the number of the cells between 3xTg-AD and control animal, neither in Sham-operated nor in OVX groups (**Figure 10C**). However, the AChE fiber density was significantly decreased in 3xTg-AD animals [*F*_(1,22)_ =29.49, *p*< 0.0001], with a significant interaction between genotype and OVX [*F*_(1,22)_ = 11.61, *p* = 0.0025]. In 3xTg-AD mice, OVX surgery exacerbated the fiber loss compared to the Sham group (*p* = 0.0147) (**Figures 10B, D**).

## 4 Discussion

In contrast to our hypothesis, OVX did not aggravate the appearance of AD-related symptoms in the cognitive behavioral tests, but in morphological examinations, signs of neurodegeneration were visible (see amyloid deposition in the BLA, and cholinergic fiber density in the SSC). **Table 3** contains the summary of the changes.

**Table 3:** Summary table of the main effect of genotype, OVX surgery, and interaction between the two parameters in the different procedures.

Category	Parameters	3xTg-AD	OVX	Interaction
Somatic	Body weight	↑	↑	Ø
	Fat	↑	↑	Ø
	Lean	↓	↓	Ø
	Uterus	Ø	↓	Ø
	Z-score	Ø	↑	+
Cognitiv	Short term in Y-maze	Ø	Ø	Ø
	SD	Ø	Ø	Ø
	MWM	Ø	Ø	Ø
	OC	↓	Ø	Ø
	CFT: freezing	↑	Ø	+
	Z-score	Ø	Ø	Ø
Anxiety	EPM: open arm time	Ø	↓	+
	EPM: open arm preference	Ø	Ø	+
	LD	Ø	Ø	Ø
	Fox odor	↑	Ø	Ø
	Z-score	↑	Ø	+
Locomotor	Y-maze	↓	Ø	Ø
	OF	↓	Ø	Ø
	EPM	(↓)	Ø	Ø
	LD box	↓	Ø	Ø
	Fox odor	(↓)	Ø	Ø
	Z-score	↓	Ø	+
Social interaction	Sociability	Ø	Ø	Ø
Morphology	Amyloid-β	N.M.	↑	N.M.
	ChAT cell number	Ø	Ø	Ø
	AChE fiber density	Ø	↓	+

Up arrow ↑—increased, Down arrow ↓—decreased, Ø—no effect, +—positive interaction, ()—tendency, N.M. not measured.

We confirmed that our model worked, as OVX induced the expected increase in body weight with fat accumulation as well as decrease in uterus weight and lean body percentage. The lack of sexual steroids can cause an increased risk for obesity, since E2 and PG also mediate glucose and lipid metabolism, and also affects adipocyte physiology (54, 82, 83). Indeed, in human studies, an increased visceral fat mass can be seen on women after menopause (84, 85). This is supported in mice by our MRI findings, where the body fat ratio of the OVX groups increased. Importantly, obesity is a prominent risk factor for AD: increasing Aβ plaques, adipokines, and cytokines, and effecting insulin homeostasis [reviewed in (86–88)]. Thus, this might be associated with how OVX might aggravate the development of AD-like symptoms. In support, 3xTg-AD animals per se were fatter and greasier, suggesting—together with the OC data—some metabolic disturbances, which require further studies. In contrast, after OVX, the weight of the uterus decreased, which can be explained by the estrogen deficit. Indeed, E2 has a proliferative effect on the uterus; hence, its lack causes hypotrophy (55, 89, 90). In future studies, luteinizing hormone measurements can help better understand the effect of OVX on the hypothalamic–hypophyseal–gonadal axis and their role in the development of AD (91–93). The MRI data also showed a decreased body lean ratio in the OVX groups, which may be the prodrome of a most common problem in menopausal patients, osteoporosis. Indeed, female sex hormone depletion was linked closely to low bone mineral density (94). Estrogen receptors can be also found in the bone, mediating protection of the bone structure, by inhibiting osteoclast activity and stimulating development of long bones and pubic epiphyses (94–96). The OVX-induced somatic changes presented in the literature were also supported by the somatic z-score, calculated from the body weight change, body fat ratio, and uterus weight. Interestingly, the OVX-induced changes were smaller in 3xTg-AD mice (see genotype × OVX interaction in somatic z-score).

As the major symptom of dementia is the cognitive decline, we evaluated five different memory tests to have a comprehensive picture. They measure different modalities [spontaneous exploration (Y-maze), social stimulus, simple association-based reward (OC) or punishment (CFT), or even complex association based on spatial memory (MWM)]. The cumulative effect (z-score) was very similar to the single tests, with overall ineffectiveness of the genetic deletion in the 3xTg-AD animals as well as the OVX. According to the literature, 3xTg-AD animals develop memory loss after 6 months (36, 38). Hence, for our animals that were between 4 and 5 months old, the results are not unexpected. However, we could not support our hypothesis, as the OVX did not aggravate the cognitive decline (no OVX effect was detected whatsoever). Even the tendencies for learning impairment in MWM were detected separately for OVX and AD without any interaction. The only genotype × OVX interaction in cognition was seen during the CS-induced freezing in CFT, when OVX aggravated the symptoms in Control, but decreased in 3xTg-AD animals. Although we used CFT as an associative learning and memory test (97), its result strongly depends on the animal’s anxiety state (98). Indeed, these CFT results were very similar to the anxiety z-score data. The intact memory can also be explained by the lack of Aβ deposition in the hippocampus and cortical areas (99, 100). We might assume that more time is needed for the development of the symptoms; therefore, investigating memory deficit would be informative with older animals only even after OVX (101).

Anxiety is a core symptom of postmenopausal women (102), as well as might be comorbid with AD (103). However, anxiety symptoms remain largely unexplored, despite the significant impact on quality of life, if not diagnosed and treated (102). As anxiety is associated with both AD and OVX (23), we assumed that both interventions will increase its level in mice, with a possible synergistic effect. However, we found a significant anxiogenic effect of OVX in EPM only, the most frequently used anxiety test (64, 104). On the contrary, an AD effect was visible in the FOT test measuring innate fear and anxiety-related behavior (75). We found that 3xTg-AD animals spend more time freezing, which suggests that these animals were more frightened (75, 105). Also, 3xTg-AD animals spend less time exploring and rearing, which might reflect anxiety, too (see immobility in CFT) (106). Nevertheless, these findings may be related to the increased Aβ deposition in the BLA (**Figure 9**), as this region is responsible for formation of fear-related responses and can be linked to anxious behavior (105, 107–109). The increased overall anxiety z-score of 3xTg-AD animals coincides with the increased anxiety in human AD patients (110, 111).

Moreover, the locomotor activity shown by the different behavioral tests (distance moved in EPM, OF, and FOT; total number of entries in the Y-maze; and number of entries to closed arms or dark compartment in EPM and LD) and the locomotion z-score calculated from these parameters showed a difference between the two genotypes with lower levels in 3xTg-AD animals. In line with previous results, this decreased locomotor activity may reflect anxious behavior. However, we cannot close out a moderate motoric disabilities as well (112, 113). The decrease in movement can be related to the presence of Aβ in the motoric and somatosensory cortex (**Figure 9**) (114). Nevertheless, in line with an anxious phenotype, OVX also decreased locomotion, which was mainly detectable in controls. In support, volcano mice presented a scalloped pattern of daily activity during the estrous cycle and OVX reduced the total movement (115). Moreover, in estrogen receptor knockout mice (on C57BL6 background), E2 injection to OVX animals increased total activity and amplitude (116). The smaller effects in the AD model might be due to the already low levels, which cannot be easily decreased further.

Despite subtle behavioral changes, morphological changes were more equivocal. Namely, Aβ plaques, one of the most characteristic morphological changes of AD (99, 117), appeared only in 3xTg-AD animals; however, we could detect their presence already around 5 months. Although we expected that OXV alone will lead to the appearance of pathological hallmarks in control animals, in humans, OVX induced behavioral and morphological changes only in the elderly or those having genetic mutations [e.g., ApoE-4 genotype (118–120)]. In line with this, OVX was able to increase the number of amyloid plaques in the 3xTg-AD animals, further increasing the translational values of our model. However, we detected changes in the BLA, but not in the HC and CTX. We have to note that in much older animals, OVX-induced Aβ formation was found also in the CTX and HC (121–123). Thus, BLA might be a sensitive area, where changes occur earlier than in other parts of the brain. It is known that stress, i.e., glucocorticoids, increases excitability of BLA, while E2 decreases it (124). Thus, in our hands, repeated testing, as a stressor, as well as E2 decline due to OVX, might have promoted the stress sensitivity of BLA (125, 126). In support of the E2 effect, the replacement of the hormone after OVX can decrease the number and density of Aβ plaques in rodents (25, 100, 121). This is also in line with human studies, where OXV patients were treated with hormone replacement therapy, resulting in no difference in Aβ deposition (120). These differences (namely, age, genetic predisposition, and hormone replacement) might be the cause of the controversy in the literature on OVX-induced amyloidosis in the brain reported to be missing by some (120, 127) or increased by others (13, 128–130). However, Palm et al., also using 3xTg-AD mice, showed no difference after E2 treatment in Aβ deposition (123), while Carroll et al. (121) used PG to reduce the p-Tau accumulation in the CA1 region of the hippocampus, subiculum, and frontal cortex.

The novelty of our study is that we included more behavioral tests and examined the cholinergic system as well. The importance of the cholinergic system in AD is outstanding, being the target of almost all the drugs in the market (131, 132). Thus, we decided to examine the cell numbers in the NBM (133), and their projections to the SSC (79). In the ChAT-positive cell numbers, no difference was found in 5-month-old mice, probably because of their young age. However, AChE-positive fiber degeneration was detected in 3xTg-AD mice and even aggravated after OVX, suggesting that axonal and dendritic degenerations start earlier than behavioral decline (114, 134).

Our study has certain limitations. First, we used standard diet, and phytoestrogens might have influenced the outcome. Next, we did not monitor the cycle, and the cyclic changes might increase variability in Sham-operated groups. Furthermore, to keep the number of used animals as low as possible, we used repeated testing, which might influence each other’s results. For some tests, more animals/group might have been required to see statistically significant differences.

All in all, we confirmed that OVX induced menopausal symptoms and removal of the sexual steroids aggravated the appearance of AD-related alterations in the brain without significantly influencing behavior. Thus, the OVX in young, 3-month-old 3xTg-AD mice might be a suitable model for testing the effect of new treatment options at the structural level, which can speed up testing (it is not necessary to wait 6–12 months for the animals to age). However, to reveal any beneficial effect on behavior, a later time point might be needed.

## Data availability statement

The raw data supporting the conclusions of this article will be made available by the authors, without undue reservation.

## Ethics statement

The animal study was reviewed and approved by Local committee of animal health and care and Baranya Country Office for Animal Welfare (PE/EA/918-7/2019).

## Author contributions

Conceptualization, DZ; methodology, SF, AS, BT, CS, CF, KB, PC, and TC; investigation, SF, AS, BT, CS, CF, KB, PC, and TC; validation, DZ; formal analysis, SF, AS, BT, CS, and CF; writing—original draft preparation, SF, AS, BT, CF, and DZ; writing—review and editing, CS, KB, PC, and TC; visualization, SF, AS, BT, CS, CF, and DZ; supervision, DZ; project administration, DZ; funding acquisition, DZ. All authors have read and agreed to the published version of the manuscript.

## Funding

This study was supported by the National Research Development and Innovation Office of Hungary (grant numbers K141934, K138763, and K120311) as well as by the Thematic Excellence Program 2021 Health Sub-programme of the Ministry for Innovation and Technology in Hungary, within the framework of the TKP2021-EGA-16 project of the Pécs of University. The agencies had no further role in study design, and in the collection, analysis, or interpretation of the data.

## Acknowledgments

We thank all the core facilities of our institute for their supportive help: the Behavioral Studies Unit for help with behavioral testing (Dr. Kornél Demeter), the Nikon Microscopy Center for help with microscopy (Dr. László Barna and Dr. Pál Vági), the Virus Technology Unit and the Medical Gene Technology Unit for help with mouse lines, and the Metabolic Phenotyping Unit (Dr Csaba Fekete) for the help by the MRI measurement.

## Conflict of interest

The authors declare that the research was conducted in the absence of any commercial or financial relationships that could be construed as a potential conflict of interest.

## Publisher’s note

All claims expressed in this article are solely those of the authors and do not necessarily represent those of their affiliated organizations, or those of the publisher, the editors and the reviewers. Any product that may be evaluated in this article, or claim that may be made by its manufacturer, is not guaranteed or endorsed by the publisher.

## Supplementary material

Supplementary Figure 1 Immunohistochemical staining (NiDAB) of Amyloid-β_1-42_ (Aβ) plaques in different brain regions of the control animals. There was no Aβ signal detectable in the brain of control animals, therefore no quantitative measurement was possible. **(A)** Representative figures based on the Paxinos Mouse Brain atlas (4th Edition) about the brain regions of interest, framed with red: Basolateral amygdala (BLA), at Bregma -1.23 mm, Motor and somatosensory cortex (CTX) at Bregma -1.07 mm and CA1 hippocampal region (CA1-HC) presented at Bregma -2.15 mm. **(B)** Representative pictures of the BLA of the control animals after Sham or OVX surgery. **(C)** Representative pictures of the CTX of control animals after Sham or OVX surgery. **(D)** Representative pictures of the HC of control animals after Sham or OVX surgery, with a close-up to a small part of the CA1 region. Scale bar: 200µm.
