# Supplementary figures and images for: Ovariectomy-induced hormone deprivation aggravates Aβ1-42 deposition in the basolateral amygdala and cholinergic fiber loss in the cortex but not cognitive behavioral symptoms in a triple transgenic mouse model of Alzheimer’s disease
Source: Front Endocrinol (Lausanne). 2022 Oct 11;13:985424. doi: 10.3389/fendo.2022.985424 (PMC9596151; doi:10.3389/fendo.2022.985424)

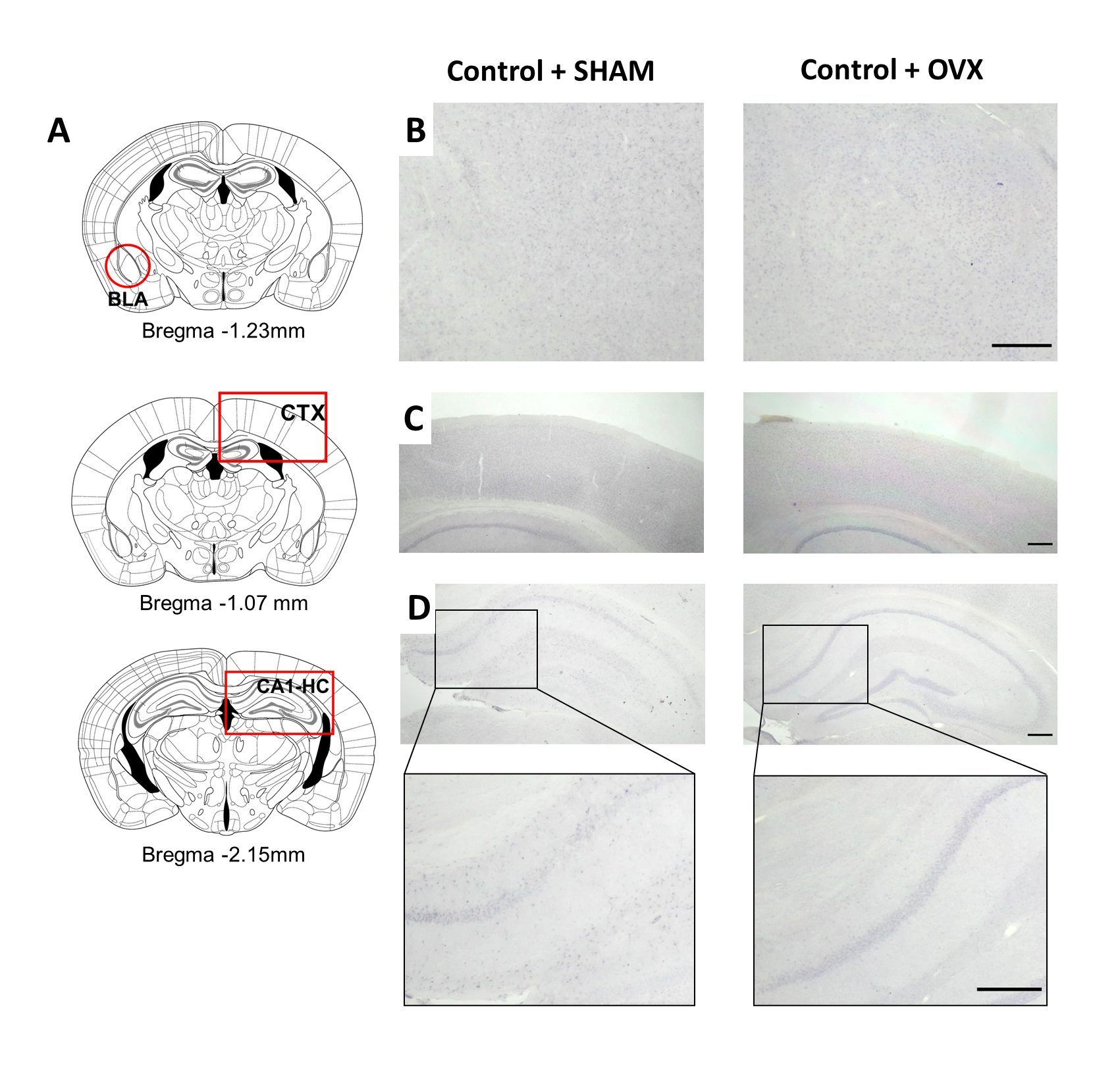

Supplement: Supplementary Figure 1 — Immunohistochemical staining (NiDAB) of Amyloid-β1-42 (Aβ) plaques in different brain regions of the control animals. There was no Aβ signal detectable in the brain of control animals, therefore no quantitative measurement was possible. (A) Representative figures based on the Paxinos Mouse Brain atlas (4th Edition) about the brain regions of interest, framed with red: Basolateral amygdala (BLA), at Bregma -1.23 mm, Motor and somatosensory cortex (CTX) at Bregma -1.07 mm and CA1 hippocampal region (CA1-HC) presented at Bregma -2.15 mm. (B) Representative pictures of the BLA of the control animals after Sham or OVX surgery. (C) Representative pictures of the CTX of control animals after Sham or OVX surgery. (D) Representative pictures of the HC of control animals after Sham or OVX surgery, with a close-up to a small part of the CA1 region. Scale bar: 200µm. [file Image_1.tif]
